# Spatial prediction of risk areas for vector transmission of *Trypanosoma cruzi* in the State of Paraná, southern Brazil

**DOI:** 10.1371/journal.pntd.0006907

**Published:** 2018-10-26

**Authors:** Andréia Mantovani Ferro e Silva, Thadeu Sobral-Souza, Maurício Humberto Vancine, Renata Lara Muylaert, Ana Paula de Abreu, Sandra Marisa Pelloso, Maria Dalva de Barros Carvalho, Luciano de Andrade, Milton Cezar Ribeiro, Max Jean de Ornelas Toledo

**Affiliations:** 1 Postgraduate Program in Health Sciences, Health Sciences Center, State University of Maringá, Maringá, Paraná, Brazil; 2 Spatial Ecology and Conservation lab (LEEC), Department of Ecology, Institute of Biosciences, São Paulo State University, Rio Claro, São Paulo, Brazil; 3 Department of Nursing, Health Sciences Center, State University of Maringá, Maringá, Paraná, Brazil; 4 Department of Medicine, Health Sciences Center, State University of Maringá, Maringá, Paraná, Brazil; 5 Department of Basic Health Sciences, Health Sciences Center, State University of Maringá, Maringá, Paraná, Brazil; Yale University, UNITED STATES

## Abstract

After obtaining certification of the absence of transmission of the *Trypanosoma cruzi* by *Triatoma infestans* in 2006, other native species of protozoan vectors have been found in human dwellings within municipalities of the State of Paraná, Southern Brazil. However, the spatial distribution of *T*. *cruzi* vectors and how climatic and landscape combined variables explain the distribution are still poorly understood. The goal of this study was to predict the potential distribution of *T*. *cruzi* vectors as a proxy for Chagas disease transmission risk using Ecological Niche Models (ENMs) based on climatic and landscape variables. We hypothesize that ENM based on both climate and landscape variables are more powerful than climate-only or landscape-only models, and that this will be true independent of vector species. A total of 2,662 records of triatomines of five species were obtained by community-based entomological surveillance from 2007 to 2013. The species with the highest number of specimens was *Panstrongylus megistus* (73%; n = 1,943), followed by *Panstrongylus geniculatus* (15.4%; 411), *Rhodnius neglectus* (6.0%; 159), *Triatoma sordida* (4.5%; 119) and *Rhodnius prolixus* (1.1%; 30). Of the total, 71.9% were captured at the intradomicile. *T*. *cruzi* infection was observed in 19.7% of the 2,472 examined insects. ENMs were generated based on selected climate and landscape variables with 1 km^2^ spatial resolution. Zonal statistics were used for classifying the municipalities as to the risk of occurrence of synanthropic triatomines. The integrated analysis of the climate and landscape suitability on triatomines geographical distribution was powerful on generating good predictive models. Moreover, this showed that some municipalities in the northwest, north and northeast of the Paraná state have a higher risk of *T*. *cruzi* vector transmission. This occurs because those regions present high climatic and landscape suitability values for occurrence of their vectors. The frequent invasion of houses by infected triatomines clearly indicates a greater risk of transmission of *T*. *cruzi* to the inhabitants. More public health attention should be given in the northern areas of the State of Paraná, which presents high climate and landscape suitabilities for the disease vectors. In conclusion, our results–through spatial analysis and predictive maps–showed to be effective in identifying areas of potential distribution and, consequently, in the definition of strategic areas and actions to prevent new cases of Chagas' disease, reinforcing the need for continuous and robust surveillance in these areas.

## Author summary

*Trypanosoma cruzi*, the etiologic agent of Chagas' disease (CD), circulates among humans, vectors and domestic and wild reservoirs. Frequent invasion of housing by infected triatomines may present a risk of vector transmission from *T*. *cruzi* to humans. The objective of this study was to predict the potential distribution of triatomines in the State of Paraná and to infer potential high risk areas for *T*. *cruzi* vector transmission. Five distinct species of three different genera of triatomines were collected, the majority of which were *Panstrongylus megistus* (73% of occurrences). More than half of collected specimens (54.8%) were captured intradomicile. The infection rate was 24.7%, which may represent a risk of occurrence of new cases of CD by vector transmission. Ecological Niche Models indicated suitable areas to triatomine occurrence in municipalities of Northwest, North and Northeast of Paraná state. Thus, more effective management actions are needed to ensure epidemiological surveillance of CD. *P*. *megistus* is the main species that colonizes the intradomicile, reinforcing the importance of the maintenance of surveillance for directing vector control actions of the CD in the local health systems in this and similar regions, mainly in the northern region of the State of Paraná, with the intensive participation of the community and also of municipal agents.

## Introduction

Chagas disease (CD) is an important zoonosis caused by the protozoan hemoflagellate *Trypanosoma cruzi* [1] and represents an important public health problem in Latin American countries where approximately 6–7 million people are currently infected by the parasite [2]. Between 2000 and 2013, Brazil recorded 68,206 deaths per CD, an average of 4,872 deaths per year [3]. Human infection occurs mainly through vector transmission that is, contact with excreta of infected triatomines (Hemiptera: Reduviidae) with *T*. *cruzi* [4]. In 2006, after the implementation of a vector control program, Brazil was certified as free from *T*. *cruzi* transmission by *Triatoma infestans* (Klug 1834). This species was considered the main vector of CD during the last century [5].

After the control of *T*. *infestans*, *Panstrongylus megistus* (Burmeister 1835) is considered the main vector of *T*. *cruzi* in Brazil. However, within the Brazilian Amazon, ecological niche models (ENMs) projected as main vector species *Rhodnius robustus* (Larrousse 1927) and *R*. *pictipes* (Stal 1872) [6]. In the State of Paraná, located in the Southern Brazil, the first record of *P*. *megistus* naturally infected by *T*. *cruzi* occurred in 1917, in the municipality of Jataí, located in the valley of the Tibagi river in the north of the state [7]. This species presents a wide geographical distribution, has high rates of infection by *T*. *cruzi* and, in addition, present a high ability to colonize artificial ecotopes [8]. In the State of São Paulo, near the State of Paraná, the increasing proximity of this species to human dwellings has been verified, which characterizes it as an important vector for *T*. *cruzi* transmission [9]. *Rhodnius prolixus* (Stal 1859) is among the five main vectors of *T*. *cruzi* and is native to Colombia and Venezuela, where it is considered the main vector of the parasite [10]. *Triatoma sordida* (Stal 1859) has been considered as emerging vector prevalent in peridomestic habitats, mainly associated with bird nests and frequently found to be infected by *T*. *cruzi*. Nevertheless, there is still no evidence of vector transmission of *T*. *cruzi* to humans by this species.

Another factor that may favor the emergence of cases of human infection by *T*. *cruzi* comes from the increase of natural and semi-natural areas inhabited by human population. These dwellings can overlap areas where natural cycles of *T*. *cruzi* occur, causing the emergence of artificial "niches" in homes and the maintenance of synanthropic reservoirs, such as mammals that have adapted to live inside or in close proximity to human dwellings [11].

ENMs use knowledge of the current distribution of the species, based on the known occurrences, together with environmental variables and mathematical algorithms, to infer suitable localities for species occurrence. Therefore, ENMs have become a widely used approach for predicting the potential distribution of species [12,13]. Once ENMs infer potential species distribution, this technique can be used to predict geographic patterns of disease transmission risk [14]. Several studies have demonstrated that the spatial distribution of triatomines populations is heavily influenced by climate conditions and changes in landscape level [6,12,15,16]. Higher temperature and humidity are one of the main determinants of the occurrence of triatomines. Additionally, the drier climate favors the dispersal of these insects, while the destruction of natural vegetation, such as forests, can modify species dispersal and species survival and local population persistence. However, no study has jointly assessed the landscape or climactic effects on the spatial distribution of triatomines in Southern Brazil. Currently, ENMs approaches have been applied to understand aspects of *T*. *cruzi* transmission, since these techniques are able to predict species geographic potential distribution [14].

Other species of triatomines, considered native, have invaded areas previously occupied by *T*. *infestans*, demonstrating evidence of ecological niche overlap [17]. This is the case of the State of Paraná, an ancient endemic area for CD. From 1995, *T*. *sordida*, *P*. *megistus* and *Rhodnius neglectus* (Lent 1954) were found in the intra and peridomicile, i.e. inside and outside the human dwellings respectively, in the rural area of municipalities of the Northwest and Central regions of the state, with infection rates by *T*. *cruzi* of up to 43% [7,18]. Moreover, residual foci of *T*. *infestans* are still detected in others Brazilian states and their elimination requires continuous and intensive entomological surveillance [5,19].

A higher number of synanthropic triatomines has also been observed in areas with higher seasonal temperature variation. The expansion of human-inhabited areas, including cities, and the modification of natural areas for agricultural activities and construction of human habitations, may disturb sites where natural cycles of *T*. *cruzi* population growth occur. This can lead triatomines to invade artificial ecotypes (like domiciles), exposing the population to these vectors, and also maintaining synanthropic reservoirs close to dwellings. This fact reinforces the effects of landscape modification on species dispersal and population persistence in no-native sites highlighting the need for an updated survey in order to direct vector prevention and control measures and, subsequently, reduce the risk of infestation of triatomines and human infection. The main goal of the present study was to analyze and spatially predict areas with environmental potential suitability for the presence of triatomines and, hence, the risk for the vector transmission of *T*. *cruzi* in the State of Paraná. For this, we used data from the period 2007 to 2013 in combination with ENM techniques based in a new approach that include climate and landscape variables. We hypothesize that both landscape and climate conditions influence the potential risk for vector transmission of *T*. *cruzi*. However, we expect that the combination of both group of variables are more powerful than climate-only or landscape-only models, and that this will be true independently of vector species.

## Methods

### Study design

We developed a cross-sectional, observational, ecological study using spatial analysis techniques, based on occurrence locations (i.e. geographic coordinates) of triatomines species in the State of Paraná, Brazil. It is important to point out that the transmission of CD depends on multiple factors for it to occur, in addition to the simple presence of triatomines infected with *T*. *cruzi*. Only in a few cases all the necessary conditions to transform a potential vector into an effective vector of CD are fulfilled. In addition to the population density of the vector and its susceptibility to *T*. *cruzi*, or to one of the parasite genetic lineages, the effective vector must attend to other conditions such as adaptation to human dwelling, high degree of anthropophilia, and short time between hematophagy and defecation. These factors are important in the contaminative vector transmission, but not for oral transmission, which is independent of some of these factors [6].

### Study area

**Fig 1:**
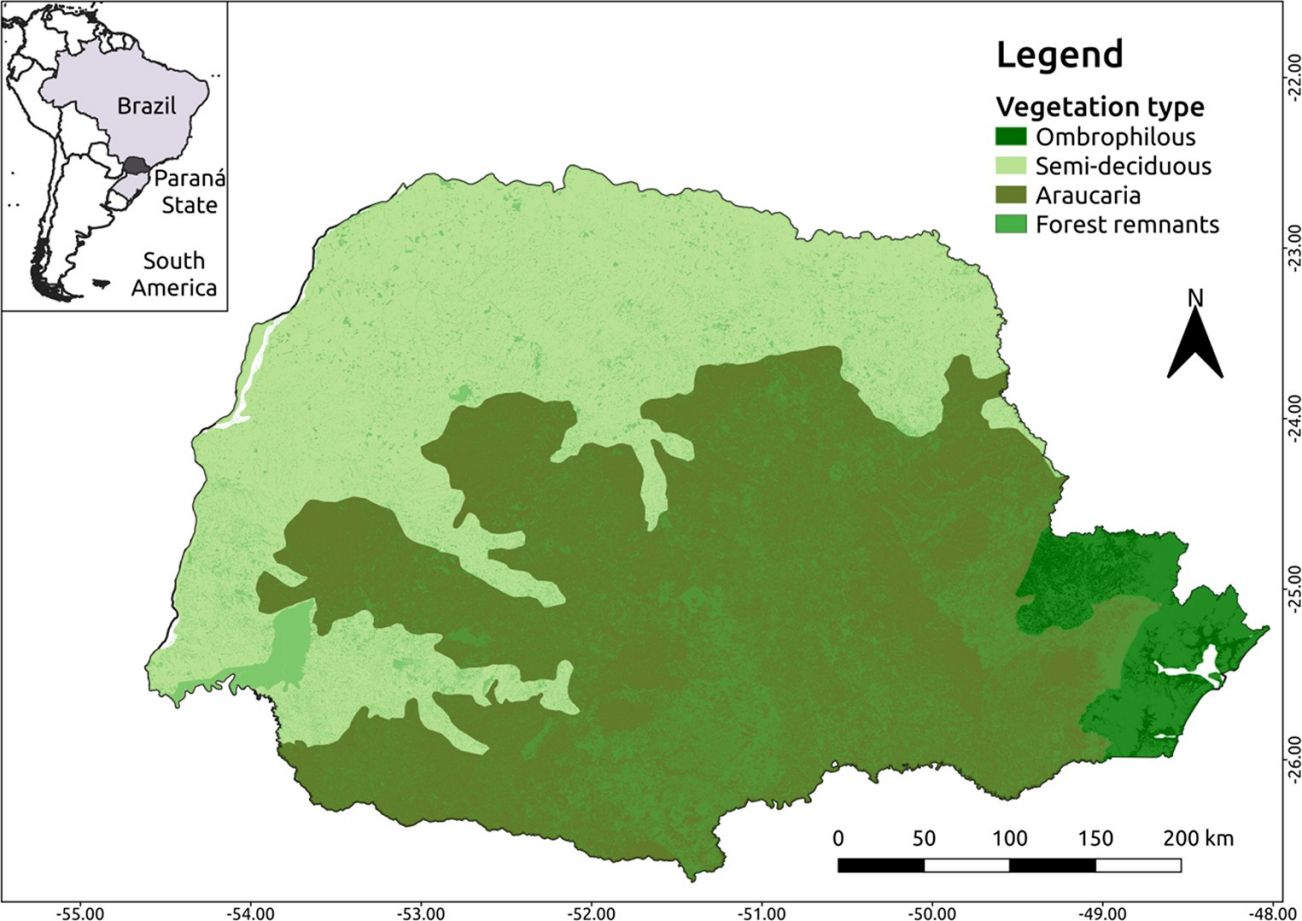
Geographical location, main vegetation types and forest remnants of the Atlantic Forest biome of the State of Paraná, Southern Brazil. Software: QGIS. Source: 1. IBGE (2015). Instituto Brasileiro de Geografia e Estatística. Continuous cartographic bases (1:250.000). Avaliable from: ftp://geoftp.ibge.gov.br/cartas_e_mapas/bases cartograficas_continuas/bc250/versao2017/. 2. Remnants: Ribeiro, M. C. et al. ATLANTIC SPATIAL: a dataset of spatial variables from the Atlantic Forest of South America (in prep.). Author: Maurício Humberto Vancine.

The State of Paraná is located in the Southern Region of Brazil in the Southern Plateau region (Fig 1), between 22°29'33'' and 26°42'59'' of latitude south, and 48°02'24'' and 54°37'38'' of longitude west, covering an area of ~200 thousand km^2^. Currently the state is subdivided into 399 municipalities with an estimated population of more than 11 million people in the year 2016 [20].

Although it covers only 2.5% of the country area, Paraná has in its territory the majority of the main phytogeographic units that occur in the country. It presents 97.8% of its territorial area inserted in the Atlantic Forest biome [21], where five large phytogeographic units stand out. (1) The Atlantic Ombrophylous Forest region is located in the eastern portion of the state that is bordered by the natural geographic barrier of the Serra do Mar. The west of this barrier, occupying the plateau portions of the state are the (2) Mixed Ombrophilous Forest with Araucaria [22]. In the northern and western regions of the state there is (3) Semideciduous Seasonal Forest (seasonal forest), characterized by two periods of climatic influence (rain and drought), partially losing its leaves, and harboring a rich biodiversity. Its original distribution occupied 37.3% of the state area and, nowadays, there are only remnants in 3.4% of it (Fig 1). (4) Areas of Estepe (Campos), cover about 14% of the state's surface, located generally in the higher portions of the three plateaus of the State of Paraná, and (5) *Cerrado*, located in the north and northeast, occupying about 1% of its area [22].

The state presents favorable climatic conditions for the development of several types of forest vegetation, which is mainly determined by the uniformity in the rainfall distribution during the year and absence of a clearly defined dry season. Following the Köeppen classification [23], the predominant climate of Paraná state is the Cfa, subtropical moist mesothermic, with average temperatures in the coldest month below 18°C and average temperature in the warmest months above 22°C, with hot summers and frosts infrequent, extending between the Paranapanema and Paraná rivers until reaching regions with altitudes between 600 and 800 m a.s.l. The highland and plateau regions present a humid subtropical climate (Cfb) with average temperature in the coldest month below 18°C, with fresh summers, and average temperature in the warmer month below 22°C. Already in the northwest region of the state, the climate is tropical modified by altitude (Cfah).

### Triatomines data sources

The occurrence data of the synanthropic triatomine species of the State of Paraná were obtained during the activities of entomologic surveillance of Chagas disease and provided by the State Department of Health of Paraná / Division of Vector-borne Diseases based on captures in domiciliary environments between 2007–2013. The use of the term synanthropic refers to the species of triatomines that frequently invade human habitations or home annexes and eventually colonize these environments [24]. The triatomines were captured through active research conducted by health agents and with the participation of community members who reported the presence of a suspected insect in their houses to health workers. The technical staff was trained to visit the house, to capture and performed a full entomological evaluation. The insects were confirmed as triatomines at the species level and submitted to examination of intestinal contents to identify positivity for *T*. *cruzi* using optical microscopy, according to the Southern Cone Initiative protocol [25].

The entomological indicators recommended by *Organización Panamericana de La Salud* and considered in this study were: number of insects captured per municipality, catch site (intra or peridomiciliary) and rate of natural infection. As the occurrence data are derived of municipality health departments, all triatomine species occurrence are associated with a central geographic coordinate that refers to the municipality centroid.

### Prediction of the risk area of Chagas' disease in the State of Paraná

The ENM approach was used to achieve the proposed objectives. The data of the bioclimatic variables were obtained from WorldClim dataset v. 1.4 (www.worldclim.org). The landscape variables were the same used by Jorge et al. (2013) [26,27]: vegetation cover (%), structural connectivity (in log (ha)*100), functional connectivity to 200 m (in log (ha)/100), Euclidean Distance to the nearest road and Human density (hereafter Anthropogenic Distance), and homogeneity from Earthenv (http://www.earthenv.org/texture) [28]. We choose functional connectivity to 200 m because we agree that it is an interesting value considering the movement on triatomines in the landscape All variables were used with spatial resolution of 1 km^2^ for Atlantic Forest delimitation and cropped to our study area, State of Paraná, entitled Political-Administrative Division of the State of Paraná in the year of 2010 [20]. These variables were used in models building because is known the effects of landscape metrics in species dispersal, species survival and population persistence [29], factors that increase the needs of use of landscape metrics to predicting transmission risk of Chagas disease.

Initially, we used all 19 bioclimatic variables and two relief variables (S1 Table) and five landscape variables (S2 Table). A factorial analysis was performed, similar to the methods applied by Sobral-Souza et al. [28], to select the climatic variables that were less correlated with each other and which explained the greater environmental variation of the studied area. The selected climatic variables for the construction of climate-based models were isothermality, temperature annual range, mean temperature of warmest quarter, precipitation of wettest quarter and precipitation of coldest quarter (S1 Table). The landscape variables used are related of the effects of landscape fragmentation in the triatomine species dispersion, since triatomines are associated with human occupations. The type of vegetation cover of an area, the plant species, its status of preservation and diversity of species, may influence the occurrence and abundance of certain species of triatomine vectors. Thus, we used vegetation cover and functional connectivity as variables for the construction of landscape models (S2 Table).

**Fig 2:**
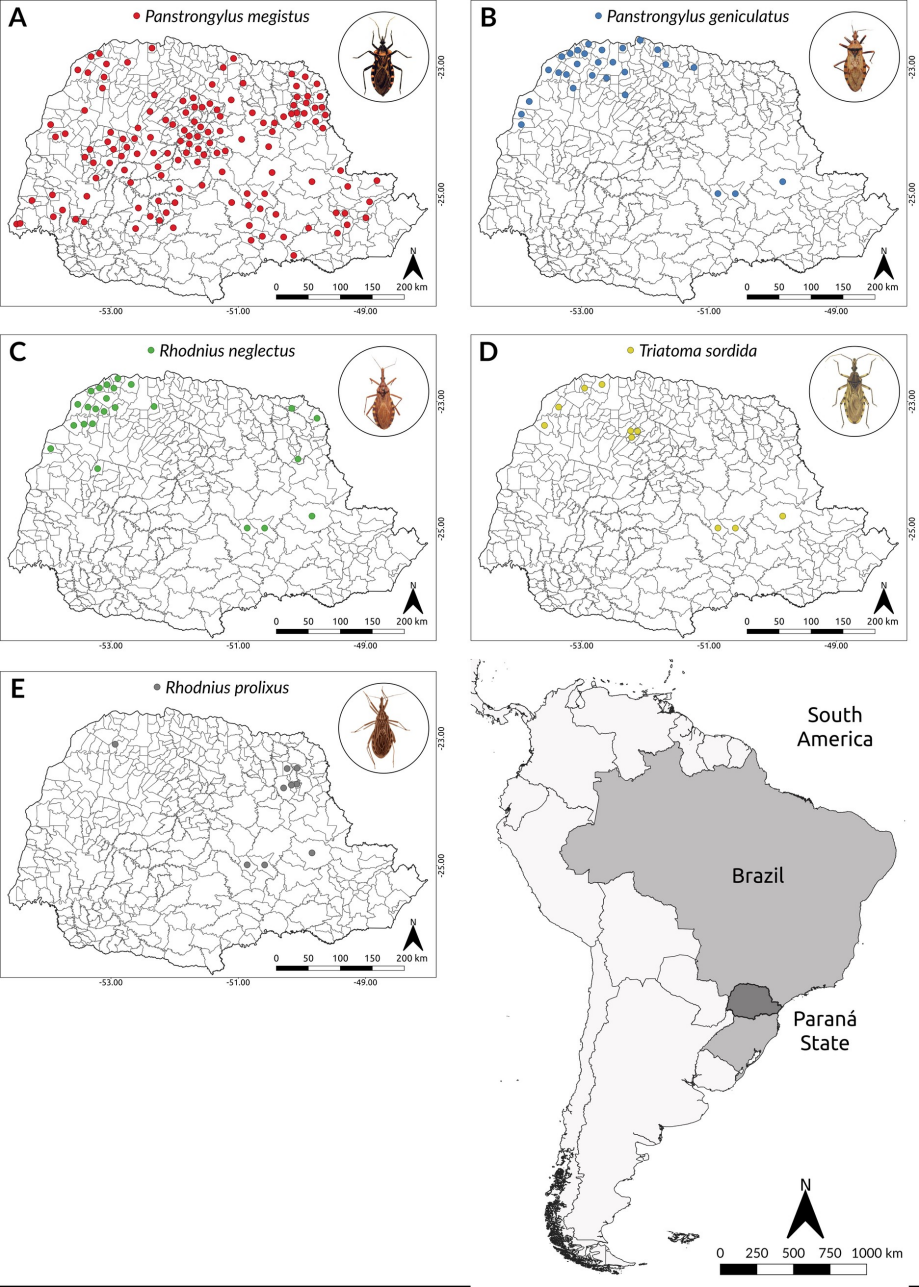
Occurrence locations of synanthropic triatomines species captured from 2007 to 2013 in the State of Paraná, Southern Brazil. A. *Panstrongylus megistus*; B. *P*. *geniculatus*; C. *Rhodnius neglectus*; D. *Triatoma sordida*; and E. *R*. *prolixus*. Software: QGIS. Source: 1. IBGE (2015). Instituto Brasileiro de Geografia e Estatística. Continuous cartographic bases (1:250.000). Avaliable from: ftp://geoftp.ibge.gov.br/cartas_e_mapas/bases cartograficas_continuas/bc250/versao2017/. 2. Occurrences: Brasil Ministério da Saúde, Secretaria de Vigilância em Saúde. Doença de Chagas aguda no Brasil: série histórica de 2000 a 2013. Boletim Epidemiológico. 2015;46: 21. Author: Maurício Humberto Vancine.

**Fig 3:**
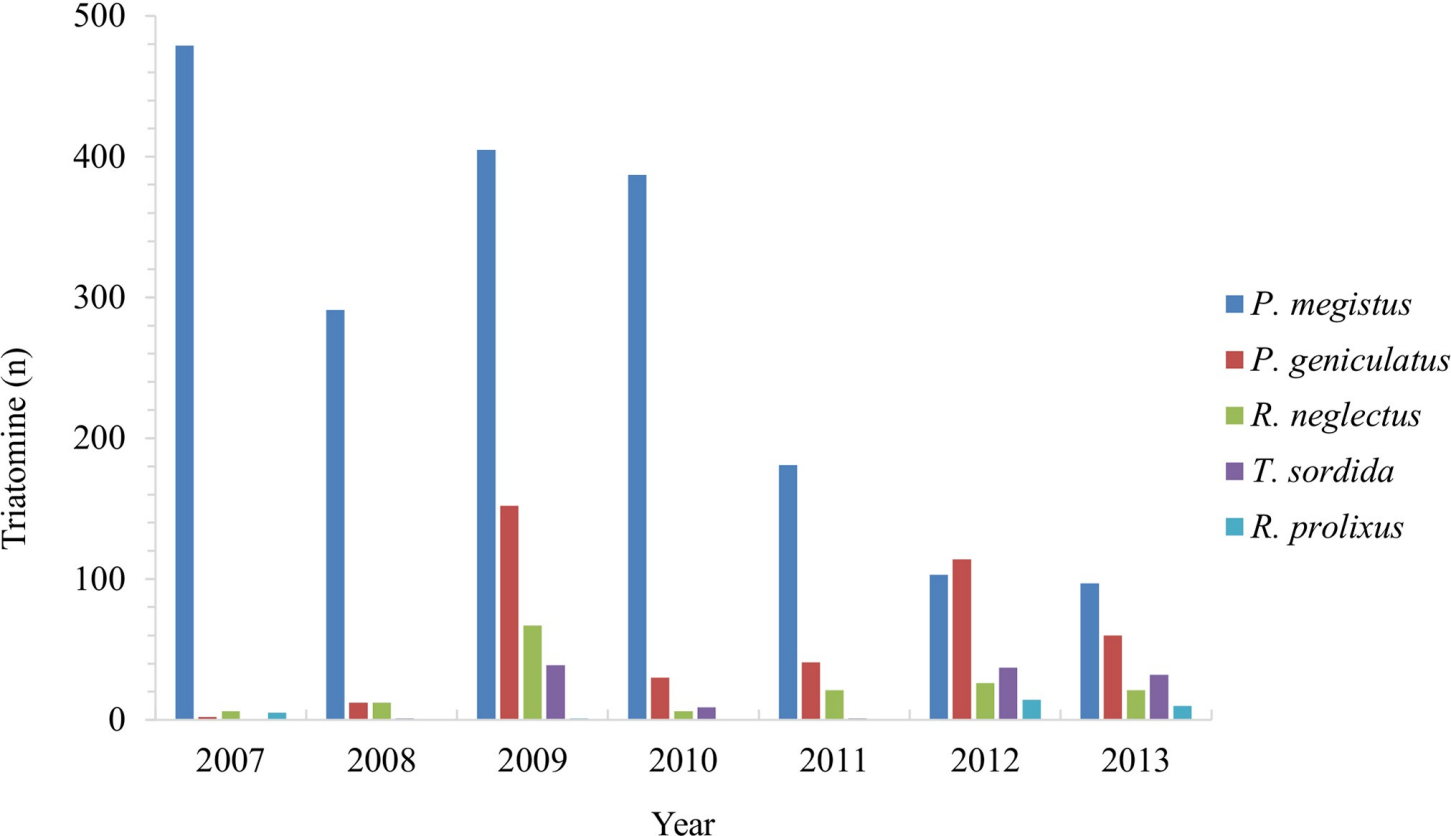
Number of triatomine specimens captured and their abundance during the study period in the State of Paraná, Southern Brazil.

To build ENMs, the values of each selected variables were extracted cell by cell using 1 km^2^ as cell-size resolution. Knowing that distinct triatomine species are listed as potential vectors, even differing as to its importance in the epidemiology of Chagas disease, all occurrence points gathered regardless of species or sampling date. Thus, the occurrence locations used in the ENMs are related to the five most frequent species in the State of Paraná during the study period (Figs 2 and 3).

Different analytical methods are available to infer the geographical distribution of species. Based on idea that the combined use of different mathematical algorithms increase the accuracy of the prediction results by considering different tolerances in the potential distribution of the species [30, 31], four mathematical algorithms were used that belong to two classes of models: two algorithms that consider only presence, represented by the climatic envelope—Bioclim [32] and by the distance method—Domain Gower Distance [33]; and two algorithms of presence/background: Support Vector Machines (SVM) [34] and Maximum Entropy (MaxEnt v. 3.3.3k) [35].

To evaluate the generated models, the occurrence points were divided into two subsets, training and test, which contained 75% and 25% of the occurrence locations, respectively. Since these subsets are part of the same set of data (points of occurrence), this process was repeated 10 times, using the k-folding (k = 2) technique, as a way of decreasing the autocorrelation between the data [13]. Thus, 40 different predictions were generated (10 randomizations x 4 algorithms) for climate-based models, and another 40 predictions for landscape models, separately. The threshold values of each model were calculated as a way of transforming them in binary maps, from the maximum specificity and sensitivity. This threshold was used because it maximizes the correctness of presences and absences and has shown to be more efficient in predicting occurrences based on presence-only models previously used [36].

After defining the thresholds, the ensemble forecasting technique [30,31] was used to obtain the final prediction map of the triatomine distribution. The maps were generated based on climate and landscape separately. We binarized the 10 maps belonging each algorithm (replicates) by their respective threshold values previously calculated and than summed the maps of the same algorithm and between the algorithms. Thus, the suitability values of each cell in the final maps varied from 0 to 40, demonstrating the frequency with which each cell was predicted as occurrence for the triatomines.

In order to evaluate each of the generated models, the values of TSS (True Skill Statistic) were estimated. The values of TSS vary from -1 to 1, where negative values or close to 0 indicate that the models do not differ statistically from randomly generated models; values close to 1 indicate excellent models, and values above 0.5 are considered as adequate models [37].

The final distribution maps of triatomines, both climate-based and landscape-based, were of 1 km^2^ cell-size resolution. However, as the objective of this study is to understand the risk of transmission per municipalities, we used the ENMs in 1 km^2^ as input and suitability average per municipality using zonal statistics. Thus, we built two suitability maps per municipality: climate-based and landscape-based map.

**Fig 4:**
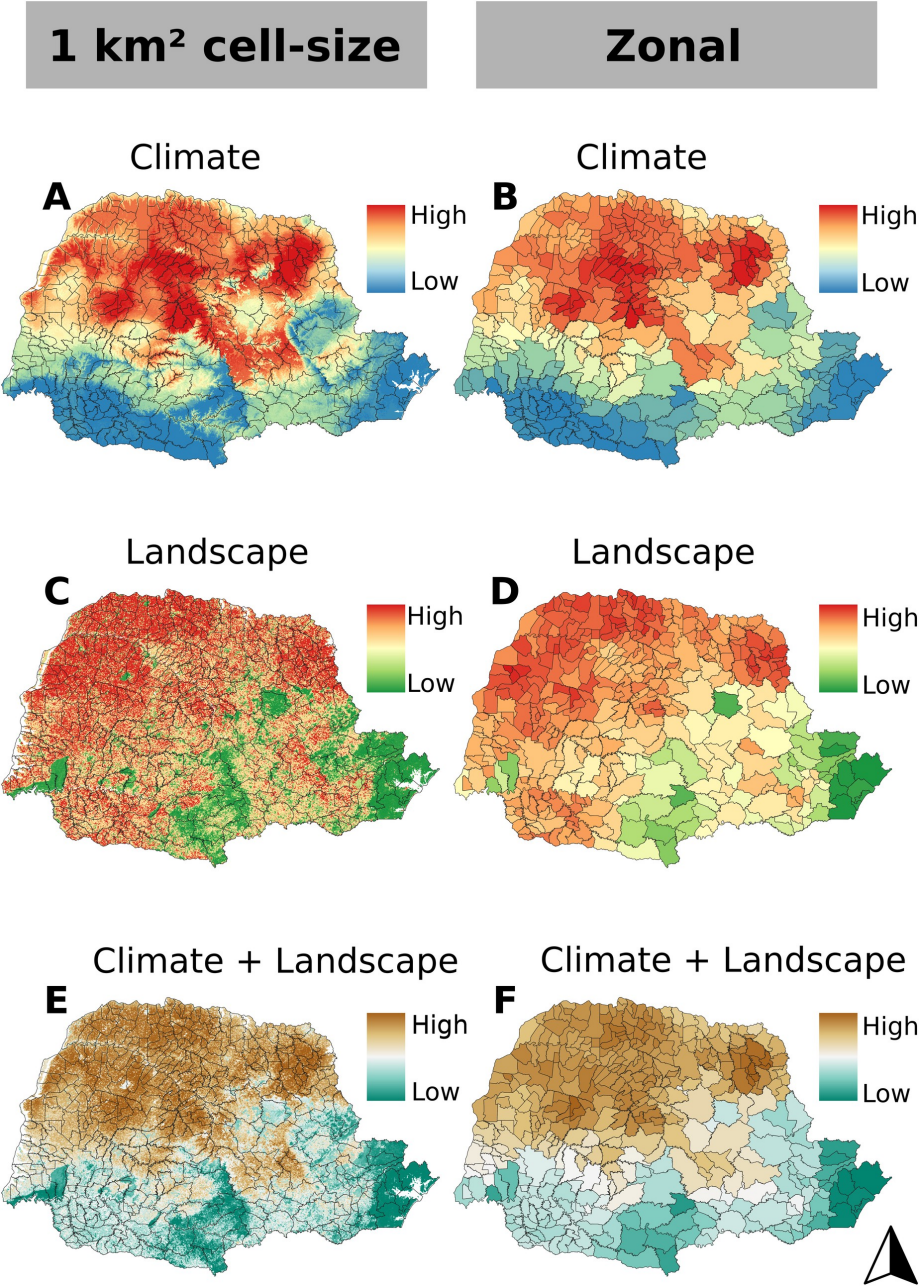
Suitability for the occurrence of triatomines within municipalities of the State of Paraná predicted using climatic and landscape environmental layers. A. Climate only; C. Landscape only; and E. Climate and landscape, using 1 km^2^ as the cell-size resolution. B. Climate only; D. Landscape only; and F. Climate and landscape, using the using zonal statistics. Software: R. Source: IBGE (2015). Instituto Brasileiro de Geografia e Estatística. Continuous cartographic bases (1:250.000). Avaliable from: ftp://geoftp.ibge.gov.br/cartas_e_mapas/basescartograficas_continuas/bc250/versao2017/. Author: Maurício Humberto Vancine.

First, niche models based on climatic variables were constructed as a way of inferring the influence of the climate on distribution of triatomine species in the municipalities of the State of Paraná. Likewise, models based on landscape variables were also generated, as a way to predict the effect of the landscape on the distribution of species. We built ENMs separately because landscape and climate condition affects species occurrence in different scales (narrow and broad-scale ecological processes). Finally, we generated a scatterplot with climate municipality suitability values (axis X) and landscape municipality suitability values (axis Y) to infer which municipalities in the State of Paraná have high climatic and landscape suitability for the occurrence of triatomines, municipalities with high climatic adequacy and low landscape suitability, municipalities with high landscape adequacy, but low climatic suitability, and municipalities with low climatic and landscape suitability (Fig 4). Here we proposed this new method to combine the effects of landscape and climate on species distribution, hereafter *EcoLand Analysis*.

**Fig 5:**
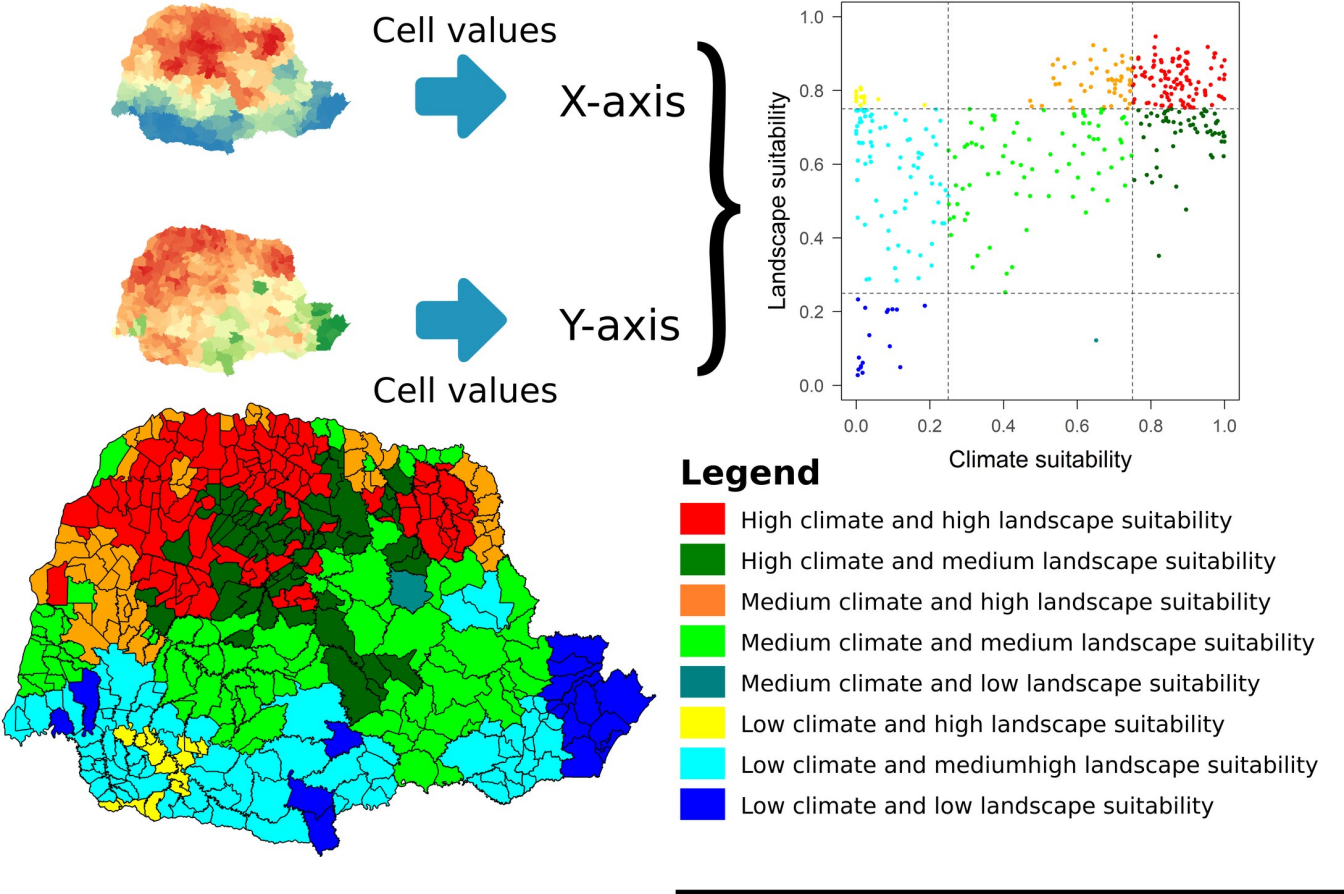
Distribution of triatomines according to climatic and landscape conditions for municipalities within Paraná state, Brazil. Software: R Source: IBGE (2015). Instituto Brasileiro de Geografia e Estatística. Continuous cartographic bases (1:250.000). Avaliable from: ftp://geoftp.ibge.gov.br/cartas_e_mapas/basescartograficas_continuas/bc250/versao2017/. Author: Maurício Humberto Vancine.

We used a 0.75 suitability threshold value for considering that a municipality is suitable or not for species occurrence (see Fig 5). Thus, the final maps of the distribution of triatomines weighs the effect sizes of climatic and landscape conditions separately.

### Ethic statement and survey permits

The State Environmental Surveillance Center (CEVA) of the State Department of Health of Paraná (SESA) (Protocol 13.669.712–9) authorized data collection of notifications and localities of triatomine species.

## Results

During the study period, a total of 2,662 specimens of triatomines belonging to five species were captured in the State of Paraná. No distinction between developmental stages was available. The species with the highest number of occurrences was *P*. *megistus* (73.0%; n = 1,943) of the specimens, followed by *P*. *geniculatus* (15.4%; 411), *R*. *neglectus* (6.0%; 159), *T*. *sordida* (4.5%; 119) and *R*. *prolixus* (1.1%; 30) (Table 1). Of the total number of insects captured, 71.9% (1,914) were found within houses, with the species *P*. *megistus* corresponding to 54.8% (1,461) of the specimens. The rate of infection by *T*. *cruzi* was 19.7% in 2,472 triatomines examined and *P*. *megistus* had the highest infection rate (24.7%).

**Table 1:** Infection rates for *Trypanosoma cruzi* of synanthropic triatomines captured from 2007 to 2013 in the State of Paraná.

	TC^a^		Intra		Peri		TE^b^		Positive	TI^c^
Species	*n*	(%)	*n*	(%)	*n*	(%)	*n*	(%)	*n*	(%)
***P*. *megistus***	1,943	(73.0)	1,461	(54.8)	482	(18.1)	1,885	(70.8)	466	(24.7)
***P*. *geniculatus***	411	(15.4)	250	(9.4)	161	(6.0)	317	(11.9)	15	(4.7)
***R*. *neglectus***	159	(6.0)	138	(5.2)	21	(0.8)	157	(5.9)	3	(1.9)
***T*. *sordida***	119	(4.5)	66	(2.5)	53	(2.0)	113	(4.2)	2	(1.8)
***R*. *prolixus***	30	(1.1)	-^d^	-	-	-	-	-	-	-
**Total**	2,662	(100)	1,915	(71.9)	717	(26.9)	2,472	(92.8)	486	(19.7)

^a^ Total captured.

^b^ Total examined.

^c^ Total infected.

^d^ Not detected. Intra = Intradomicile; Peri = peridomicile.

The municipalities of Paraná with the highest number of captured triatomines were Guamiranga (6.3%; 196 specimens), Rosário do Ivaí (5.6%; 148), Querência do Norte (4.4%; 118), Santana do Itararé (4.3%; 115) and Nova Londrina (3.5%; 94) (S3 Table). The triatomines were captured in 39.9% (159/399) of the municipalities and in 47.8% (76) of them, there were positive insects for *T*. *cruzi*, and in 13.2% (21) of the municipalities, the insect infection rate was 100%.

Between 2007 and 2013, the number of insects caught varied between 492 and 223, with a peak of 664 specimens in the year 2009. *P*. *megistus* prevailed in 6/7 years (except for 2012 when *P*. *geniculatus* was the most captured), showing a decline, so much that in 2007 that species corresponded to 97.4% of the insects caught and in 2013 44.1% (Fig 3).

All generated models had TSS values above than 0.5 (S4 Table). The results of the predictions show that in the analysis of 1 km^2^ cell-size and in the zonal model (which demonstrates the mean values for each municipality), the northern and central regions of the state, identified in red on the map, present the most suitable areas for the presence of triatomines species (Fig 4A and 4B). The southern and eastern regions, highlighted in blue on the map, have areas with low potential for the presence of triatomines species. The most suitable climate for the occurrence of triatomines is the hot and humid and the optimum levels for most species are around 26–29°C and 70% relative humidity [38].

On the other hand, the main physiographic regions where occur four of the five triatomines species found in this study are tropical and subtropical, dry and xerophytic forests (i.e. develop in areas with low humidity and is the main representatives of the Subtropical Forests of southern Brazil) and, to a lesser extent, savannas [39]. The landscape-based predictions show a rather different distribution pattern, with all the northern extension and part of the western part of the state being considered suitable for triatomine occurrence (Fig 4C and 4D). When comparing climate and landscape prediction maps, it can be observed that most municipalities appear in red, indicating areas with high suitability for the occurrence of triatomines species. However, some blue areas on the climate map were orange in the landscape map (medium suitability).

The *EcoLand* results, combining climate and landscape effects on triatomine occurrence indicated that the northern and northwestern regions are more suitable for the occurrence of triatomine species (Fig 4E and 4F). The scatterplot (climate suitability x landscape suitability) results indicates that there are municipalities with a high suitability for the occurrence of triatomines, both for landscape and by climate conditions (Fig 5). However, it has been identified that many municipalities have either adequate climate or landscape and other locations have low suitability for both climate and landscape. It is noticeable that some municipalities in the northwestern, northern and northeastern of the state (marked in red on the map) have a greater risk of *T*. *cruzi* vector transmission due to the high climatic and landscape suitability for the occurrence of their vectors (S1 Table). Besides these, a subset of others conditions under which the vector is able to survive and reproduce in must also be met. Of the 399 municipalities analyzed, 26% (n = 104) were classified as having high climate and landscape suitability, and only 4% (16) had low climate and landscape suitability for the occurrence of the vectors.

## Discussion

During the seven years of study, five species of synanthropic triatomines were captured in close to 40% (159/399) of the municipalities of the State of Paraná. Most of the entomological data came from passive surveillance and 72% of captures occurred in the intradomicile. *P*. *megistus*, strongly associated with humid regions, was the most prevalent species, it had the highest rate of infection by *T*. *cruzi* (24.7%), about five times higher than the other species, and most of the specimens were found in the intradomiciliary (54.8%). This species therefore should receive more attention from the health authorities, adopting stricter entomological surveillance.

Previous studies showed *T*. *sordida* as the species most frequently captured in Paraná, usually with high infection rates for *T*. *cruzi* in the peridomicile [7,18]. However, that was not the case in the present study, since *P*. *megistus* was the most frequently captured species. Moreover, *T*. *infestans* were not found, confirming the efficacy of the Southern Cone Initiative to Control Chagas Disease program in the state [40]. *P*. *geniculatus* was the second most captured species in the study period, overcoming *P*. *megistus* in 2012 in number of captured specimens—but presenting a low infection rate (4.7%). This is considered a wild species associated with armadillo holes [41], however, it has also been observed to be associated with domestic pigs in the Amazon basin in Northern Brazil [42]. Attracted by light, they are frequently found inside human dwellings and, together with *P*. *megistus*, they occur in more than 20 Brazilian states and in at least three Brazilian biomes, showing large potential for adaptation to different ecological conditions [43].

The low frequency observed of the species *R*. *neglectus* and *T*. *sordida* may have been influenced by the type of vegetation cover of the studied area. Gurgel-Gonçalves *et al*. [6] used ENMs for generate suitability maps of these species in the *Cerrado* biome, unlike the State of Paraná that, as shown previously, has only 1% of *Cerrado* in its territorial area. All *Rhodnius* species have been primarily associated with palm trees even though some species were found in other sylvatic habitats. Although *R*. *neglectus* has a wider distribution across the *Cerrado* it has also been found in the adjacent regions of Central Brazil. In the decades after the implementation of the control program that led to the elimination of *T*. *infestans*, *T*. *sordida* was the most common species in Paraná, corresponding to 87.4% and 98.7% of the captured specimens, with rates of infection by *T*. *cruzi* ranging from 13.4% to 43.0% [7,18]. The areas of higher occurrence of *T*. *sordida* are the ones related to the agricultural activities in the past, what could explain its presence in areas of the state of Paraná that suffered ecologic impact due to significant loss of vegetation. However, the results of the current study show that their abundance in this state did not hold, occupying the 4th place in terms of frequency (4.7%) with low infection rate (1.8%).

Among the five triatomine species we analyzed, *R*. *prolixus* was the least frequent (1.1% of the captured specimens), showing a slight increase in the last two years. A low frequency of this species was also observed in a study of the spatial distribution of triatomines in the State of Minas Gerais, southeastern Brazil [44]. For being considered one of the main vectors of *T*. *cruzi*, it deserves attention from the sanitary authorities to prevent the expansion in its geographical range.

Several studies on the distribution of triatomines in Brazil have considered the capacity of domiciliary or home invasion associated with climatic conditions [15] and biogeographic aspects related mainly to vegetation cover [16]. In this study, an ENM approach was used, which allows the exploration of geographic and ecological phenomena based on known occurrences of species [32]. From these techniques, risk areas can be defined by analyzing the geographical distribution of disease cases, vectors or reservoirs. In a study by Peterson et al. [45] it was possible to develop distribution hypotheses for 15 species of mammals and insects that interact in the potential transmission of *T*. *cruzi* in Mexico.

The variables isothermality, temperature annual range, mean temperature of warmest quarter, precipitation of wettest quarter and precipitation of coldest quarter, were selected for ENMs because they are less correlated with each other and explain the greater climatic variation of Paraná state. However, the subtropical humid mesothermic climate (Cfa), which predominates in the north and central regions of the state, is more suited to the triatomine encounter, differing from the climate with fresh summers and temperatures below 22 ^o^C occurring in the mountains and plateau regions. Our climate-based models predicted that all selected climate variables affect triatomine species occurrence and indicate that northwest, north and northeast regions has suit climate and landscape condition to maintain triatomine population. The southern region of Paraná state has severe climate condition (low temperature) for triatomine occurrence. The influence of temperature on triatomine distribution has been reported in literature [15,46,47,48]. Higher temperature values favor greater geographical dispersion of wild vectors [38]. Moreover, the type of vegetation cover and the structural connectivity (that is, the landscape's ability to facilitate biological flow) also influenced the prediction of species occurrence suitability. The areas of seasonal forest and, to a lesser extent, Mixed Ombrophylous Forest, were more suitable for the occurrence of triatomines. These types of vegetation contain several species of our flora considered triatomines ecotypes, such as hollow trees in arboreal habitats, palms and epiphytes [6]. Besides the variables structural and functional connectivity may favor the flow not only of triatomine species, but also of mammalian and avian species that can act as a food source of these insects, as a source of infection (wild mammalian of different orders, known reservoirs of *T*. *cruzi*).

However, as we hypothesized, models using climate and landscape effects on triatomine distribution allowed us to clearly identify the municipalities that present greater suitability for the occurrence of synanthropic triatomines and, consequently, the risk for vector transmission of *T*. *cruzi*. These municipalities are located mainly in areas of mesothermal humid subtropical climate and in the phytogeographic unit of the Atlantic Forest biome of the seasonal forest. Main efforts should concentrate where both suitability values converge, in order to apply the best control measures. On places where there is medium of both suitability values, initiatives of prevention should be applied, in order to keep the population informed and prevented against CD.

These results allow to predict the profile of municipalities on which there is a high potential risk of *T*. *cruzi* vector transmission, without the need for sampling the focal insects. These predictions generate ecological and biogeographic knowledge regarding triatomines providing information to subsidize health services, guiding the actions of control and prevention of CD in the State of Paraná. Although our results predict a reliable spatial potential risk of *T*. *cruzi*, there are some limitations. The use of pooling data from multiple vector species, and our assumption that gathering the vector species occurrence data is plausible (whether infected or not, and whether found indoors or not) should be reviewed to improve prediction of risk of *T*. *cruzi*. It is also important to differentiate the developmental stage of triatomines since the encounter of immature forms inside houses means colonization of these insects. However, to do it, we need others field expeditions to search new triatomine species in unknown and unstudied sites. With accurate spatial and temporal information about triatomine occurrence directing disease control and preventive actions will be more effective.

## Supporting information

S1 Table Summary of the factorial analysis of the climatic variables used to model the distribution of triatomines in the State of Paraná, Southern Brazil. Bold values correspond to climatic variables used for the construction of climate-based models.
(DOCX)

S2 Table Summary of the factorial analysis of the landscape variables used to model the distribution of triatomines in the State of Paraná, Southern Brazil. Bold values correspond to landscape variables used for the construction of landscape models.
(DOCX)

S3 Table Climate and landscape suitability of the 399 municipalities of the State of Paraná for the occurrence of triatomines. (DOCX)

S4 Table The True Skill Statistic (TSS) values of climate-based models and landscape-based models. We used the following algorithms: Bioclim, Gower, Maxent, and numbers from 1 to 10 are the model replicates. SD—Standard deviation.
(DOCX)
