# Supplementary material for: Spatial prediction of risk areas for vector transmission of Trypanosoma cruzi in the State of Paraná, southern Brazil
Source: PLoS Negl Trop Dis. 2018 Oct 26;12(10):e0006907. doi: 10.1371/journal.pntd.0006907 (PMC6221357; doi:10.1371/journal.pntd.0006907)
Supplement: S1 Table — Bold values correspond to climatic variables used for the construction of climate-based models. (DOCX) [file pntd.0006907.s001.docx]

**S1 Table. Summary of the factorial analysis of the climatic variables used to model the distribution of triatomines in the State of Paraná, Southern Brazil.** Bold values correspond to climatic variables used for the construction of climate-based models.

| **Environmental variable** | **Factor 1** | **Factor 2** | **Factor 3** | **Factor 4** | **Factor 5** |
| --- | --- | --- | --- | --- | --- |
| **Altitude** | 0.96 | 0.08 | 0.08 | 0.09 | 0.1 |
| **Annual Mean Temperature** | 0.95 | 0.27 | 0.08 | 0.02 | 0.11 |
| **Mean Diurnal Range** | 0.17 | 0.25 | 0.34 | 0.79 | 0.39 |
| **Isothermality** | 0.19 | 0.11 | 0.44 | 0.25 | **0.82** |
| **Temperature Seasonality** | 0.05 | 0.49 | 0.2 | 0.45 | 0.7 |
| **Max Temperature of Warmest Month** | 0.96 | 0.02 | 0.07 | 0.27 | 0.01 |
| **Min Temperature of Coldest Month** | 0.87 | 0.32 | 0.02 | 0.35 | 0.11 |
| **Temperature Annual Range** | 0.06 | 0.43 | 0.12 | **0.88** | 0.15 |
| **Mean Temperature of Wettest Quarter** | 0.83 | 0.32 | 0.04 | 0.07 | 0.01 |
| **Mean Temperature of Driest Quarter** | 0.8 | 0.14 | 0.02 | 0 | 0.33 |
| **Mean Temperature of Warmest Quarter** | **0.98** | 0.17 | 0.02 | 0.06 | 0.03 |
| **Mean Temperature of Coldest Quarter** | 0.92 | 0.31 | 0.09 | 0.1 | 0.2 |
| **Annual Precipitation** | 0.19 | 0.71 | 0.64 | 0.09 | 0.12 |
| **Precipitation of Wettest Month** | 0.03 | 0.18 | 0.92 | 0.03 | 0.13 |
| **Precipitation of Driest Month** | 0.4 | 0.81 | 0.29 | 0.18 | 0.16 |
| **Precipitation Seasonality** | 0.42 | 0.81 | 0.27 | 0.16 | 0.14 |
| **Precipitation of Wettest Quarter** | 0.04 | 0.08 | **0.98** | 0.12 | 0.12 |
| **Precipitation of Driest Quarter** | 0.34 | 0.87 | 0.25 | 0.2 | 0.12 |
| **Precipitation of Warmest Quarter** | 0.05 | 0.09 | 0.97 | 0.12 | 0.1 |
| **Precipitation of Coldest Quarter** | 0.19 | **0.92** | 0.19 | 0.2 | 0.01 |
| **Slope Declivity** | 0.19 | 0.03 | 0.19 | 0.03 | 0.13 |
