# Supplementary material for: Spatial prediction of risk areas for vector transmission of Trypanosoma cruzi in the State of Paraná, southern Brazil
Source: PLoS Negl Trop Dis. 2018 Oct 26;12(10):e0006907. doi: 10.1371/journal.pntd.0006907 (PMC6221357; doi:10.1371/journal.pntd.0006907)
Supplement: S2 Table — Bold values correspond to landscape variables used for the construction of landscape models. (DOCX) [file pntd.0006907.s002.docx]

**S2 Table. Summary of the factorial analysis of the landscape variables used to model the distribution of triatomines in the State of Paraná, Southern Brazil.** Bold values correspond to landscape variables used for the construction of landscape models.

| **Landscape variable** | **Axes 1** | **Axes 2** |
| --- | --- | --- |
| **Anthropogenic Distance** | 0.09 | 0.48 |
| **Homogeneity** | 0.2 | 0.49 |
| **Vegetation Cover** | 0.23 | **0.53** |
| **Structural Connectivity** | **0.94** | 0.31 |
| **Functional Connectivity 200 m** | 0.94 | 0.3 |
