# Supplementary material for: Spatial prediction of risk areas for vector transmission of Trypanosoma cruzi in the State of Paraná, southern Brazil
Source: PLoS Negl Trop Dis. 2018 Oct 26;12(10):e0006907. doi: 10.1371/journal.pntd.0006907 (PMC6221357; doi:10.1371/journal.pntd.0006907)
Supplement: S3 Table — (DOCX) [file pntd.0006907.s003.docx]

**S3 Table: Climate and landscape suitability (mean) of the 399 municipalities of the State of Paraná for the occurrence of triatomines.**

| **Id** | **Municipality** | **N^o^ of triatomines per municipality** | **Climate suitability** | **Landscape suitability** | **Suitability level** |
| --- | --- | --- | --- | --- | --- |
|  |  |  |  |  | **(Climate and landscape)** |
| 1 | Rancho Alegre D'Oeste | 15 | 0.98 | 0.79 | High and high |
| 2 | Guapirama | 11 | 1.00 | 0.84 | High and high |
| 3 | Inajá | 0 | 0.77 | 0.89 | High and high |
| 4 | Esperança Nova | 0 | 0.81 | 0.95 | High and high |
| 5 | Sarandi | 0 | 0.84 | 0.78 | High and high |
| 6 | Cidade Gaúcha | 0 | 0.77 | 0.86 | High and high |
| 7 | Kaloré | 11 | 0.92 | 0.77 | High and high |
| 8 | Loanda | 4 | 0.85 | 0.83 | High and high |
| 9 | Nova Esperança | 0 | 0.90 | 0.88 | High and high |
| 10 | Nova Londrina | 60 | 0.80 | 0.83 | High and high |
| 11 | Ivaté | 1 | 0.83 | 0.76 | High and high |
| 12 | Conselheiro Mairinck | 1 | 1.00 | 0.88 | High and high |
| 13 | Alto Paraná | 2 | 0.89 | 0.88 | High and high |
| 14 | Sabáudia | 0 | 0.85 | 0.75 | High and high |
| 15 | Ibaiti | 19 | 0.99 | 0.78 | High and high |
| 16 | Lobato | 0 | 0.9 | 0.83 | High and high |
| 17 | Colorado | 0 | 0.88 | 0.80 | High and high |
| 18 | Pitangueiras | 0 | 0.86 | 0.89 | High and high |
| 19 | Itaúna do Sul | 0 | 0.81 | 0.87 | High and high |
| 20 | Tomazina | 34 | 0.84 | 0.83 | High and high |
| 21 | Mandaguaçu | 0 | 0.89 | 0.77 | High and high |
| 22 | Nova Santa Bárbara | 0 | 0.89 | 0.76 | High and high |
| 23 | Barra do Jacaré | 2 | 0.83 | 0.78 | High and high |
| 24 | São João do Caiuá | 0 | 0.86 | 0.88 | High and high |
| 25 | Pinhalão | 19 | 0.89 | 0.81 | High and high |
| 26 | Ariranha do Ivaí | 10 | 0.87 | 0.83 | High and high |
| 27 | Guairaçá | 14 | 0.84 | 0.84 | High and high |
| 28 | Santo Antônio da Platina | 8 | 0.96 | 0.86 | High and high |
| 29 | Douradina | 2 | 0.81 | 0.81 | High and high |
| 30 | Indianápolis | 0 | 0.84 | 0.85 | High and high |
| 31 | Ivaiporã | 46 | 0.90 | 0.84 | High and high |
| 32 | Arapuã | 14 | 1.00 | 0.78 | High and high |
| 33 | Cruzeiro do Sul | 0 | 0.90 | 0.90 | High and high |
| 34 | Lupionópolis | 0 | 0.78 | 0.84 | High and high |
| 35 | Quatiguá | 3 | 0.95 | 0.92 | High and high |
| 36 | Santo Inácio | 0 | 0.81 | 0.88 | High and high |
| 37 | Icaraíma | 15 | 0.83 | 0.88 | High and high |
| 38 | Umuarama | 1 | 0.83 | 0.85 | High and high |
| 39 | Mamborê | 2 | 0.84 | 0.79 | High and high |
| 40 | Paranacity | 0 | 0.88 | 0.85 | High and high |
| 41 | Assaí | 0 | 0.92 | 0.75 | High and high |
| 42 | Atalaia | 0 | 0.9 | 0.87 | High and high |
| 43 | Nova Fátima | 0 | 0.91 | 0.76 | High and high |
| 44 | Maria Helena | 0 | 0.89 | 0.85 | High and high |
| 45 | Califórnia | 5 | 0.86 | 0.79 | High and high |
| 46 | Santa Inês | 0 | 0.81 | 0.92 | High and high |
| 47 | Nova Aliança do Ivaí | 1 | 0.78 | 0.84 | High and high |
| 48 | Corumbataí do Sul | 0 | 0.91 | 0.75 | High and high |
| 49 | Japira | 3 | 0.99 | 0.87 | High and high |
| 50 | Nossa Senhora das Graças | 0 | 0.88 | 0.89 | High and high |
| 51 | Uniflor | 0 | 0.90 | 0.90 | High and high |
| 52 | Abatiá | 0 | 0.92 | 0.80 | High and high |
| 53 | Santa Isabel do Ivaí | 4 | 0.76 | 0.84 | High and high |
| 54 | Planaltina do Paraná | 3 | 0.78 | 0.85 | High and high |
| 55 | Santa Amélia | 0 | 0.85 | 0.76 | High and high |
| 56 | Mariluz | 1 | 0.83 | 0.76 | High and high |
| 57 | Goioerê | 2 | 0.95 | 0.79 | High and high |
| 58 | Barbosa Ferraz | 0 | 0.97 | 0.76 | High and high |
| 59 | Tapira | 2 | 0.75 | 0.86 | High and high |
| 60 | Araruna | 1 | 0.90 | 0.86 | High and high |
| 61 | Jundiaí do Sul | 1 | 1.00 | 0.83 | High and high |
| 62 | Joaquim Távora | 3 | 0.97 | 0.89 | High and high |
| 63 | Janiópolis | 5 | 0.97 | 0.90 | High and high |
| 64 | Presidente Castelo Branco | 0 | 0.86 | 0.82 | High and high |
| 65 | Guaraci | 0 | 0.87 | 0.87 | High and high |
| 66 | Paraíso do Norte | 0 | 0.75 | 0.81 | High and high |
| 67 | Cianorte | 0 | 0.95 | 0.80 | High and high |
| 68 | Cambira | 3 | 0.90 | 0.76 | High and high |
| 69 | Terra Roxa | 0 | 0.75 | 0.82 | High and high |
| 70 | Munhoz de Mello | 0 | 0.87 | 0.91 | High and high |
| 71 | Tapejara | 0 | 0.85 | 0.81 | High and high |
| 72 | Santa Mônica | 0 | 0.76 | 0.78 | High and high |
| 73 | Jaguapitã | 0 | 0.87 | 0.82 | High and high |
| 74 | Maringá | 0 | 0.85 | 0.75 | High and high |
| 75 | Paranavaí | 7 | 0.83 | 0.83 | High and high |
| 76 | Cruzeiro do Oeste | 0 | 0.84 | 0.81 | High and high |
| 77 | Tamboara | 0 | 0.85 | 0.85 | High and high |
| 78 | Congonhinhas | 0 | 0.89 | 0.75 | High and high |
| 79 | Farol | 1 | 0.93 | 0.82 | High and high |
| 80 | Moreira Sales | 2 | 0.83 | 0.88 | High and high |
| 81 | Cruzmaltina | 9 | 0.75 | 0.77 | High and high |
| 82 | Rondon | 0 | 0.80 | 0.83 | High and high |
| 83 | Peabiru | 0 | 0.90 | 0.78 | High and high |
| 84 | Nova Olímpia | 0 | 0.81 | 0.90 | High and high |
| 85 | Rolândia | 0 | 0.85 | 0.77 | High and high |
| 86 | Bandeirantes | 0 | 0.79 | 0.77 | High and high |
| 87 | Centenário do Sul | 0 | 0.81 | 0.86 | High and high |
| 88 | Miraselva | 0 | 0.88 | 0.89 | High and high |
| 89 | Boa Esperança | 40 | 0.99 | 0.83 | High and high |
| 90 | Flórida | 0 | 0.91 | 0.82 | High and high |
| 91 | Cafeara | 0 | 0.86 | 0.89 | High and high |
| 92 | Santa Fé | 0 | 0.88 | 0.87 | High and high |
| 93 | Ribeirão do Pinhal | 0 | 0.96 | 0.82 | High and high |
| 94 | Terra Rica | 14 | 0.77 | 0.89 | High and high |
| 95 | Japurá | 0 | 0.84 | 0.79 | High and high |
| 96 | Iguaraçú | 0 | 0.88 | 0.82 | High and high |
| 97 | Astorga | 0 | 0.87 | 0.81 | High and high |
| 98 | Juranda | 1 | 0.95 | 0.79 | High and high |
| 99 | Ângulo | 0 | 0.90 | 0.83 | High and high |
| 100 | São João do Ivaí | 5 | 0.92 | 0.79 | High and high |
| 101 | Florestópolis | 0 | 0.81 | 0.76 | High and high |
| 102 | Mandaguari | 0 | 0.88 | 0.77 | High and high |
| 103 | Jaboti | 30 | 0.97 | 0.90 | High and high |
| 104 | Alto Paraíso | 17 | 0.79 | 0.77 | High and high |
| 105 | São Carlos do Ivaí | 0 | 0.85 | 0.72 | High and medium |
| 106 | Manoel Ribas | 36 | 0.93 | 0.69 | High and medium |
| 107 | Ivaí | 0 | 0.87 | 0.54 | High and medium |
| 108 | Iretama | 6 | 0.95 | 0.70 | High and medium |
| 109 | Paiçandu | 0 | 0.97 | 0.68 | High and medium |
| 110 | São Pedro do Ivaí | 0 | 0.96 | 0.69 | High and medium |
| 111 | Ourizona | 0 | 0.96 | 0.62 | High and medium |
| 112 | Lunardelli | 8 | 0.92 | 0.70 | High and medium |
| 113 | Doutor Camargo | 0 | 1.00 | 0.62 | High and medium |
| 114 | Terra Boa | 0 | 0.95 | 0.69 | High and medium |
| 115 | Guamiranga | 196 | 0.79 | 0.57 | High and medium |
| 116 | São Sebastião da Amoreira | 0 | 0.91 | 0.71 | High and medium |
| 117 | Lidianópolis | 1 | 0.77 | 0.75 | High and medium |
| 118 | Prudentópolis | 89 | 0.82 | 0.35 | High and medium |
| 119 | Marialva | 0 | 0.94 | 0.73 | High and medium |
| 120 | Jardim Alegre | 11 | 0.95 | 0.74 | High and medium |
| 121 | Rio Branco do Ivaí | 1 | 0.80 | 0.71 | High and medium |
| 122 | Bela Vista do Paraíso | 0 | 0.80 | 0.74 | High and medium |
| 123 | São Jorge do Ivaí | 0 | 0.97 | 0.62 | High and medium |
| 124 | Nova Tebas | 23 | 1.00 | 0.69 | High and medium |
| 125 | Marilândia do Sul | 8 | 0.84 | 0.72 | High and medium |
| 126 | Bom Sucesso | 0 | 1.00 | 0.68 | High and medium |
| 127 | Campo Mourão | 30 | 0.84 | 0.74 | High and medium |
| 128 | Sapopema | 4 | 0.86 | 0.65 | High and medium |
| 129 | Itambé | 0 | 0.99 | 0.66 | High and medium |
| 130 | Jandaia do Sul | 7 | 0.92 | 0.73 | High and medium |
| 131 | Luiziana | 25 | 0.82 | 0.64 | High and medium |
| 132 | Prado Ferreira | 0 | 0.88 | 0.74 | High and medium |
| 133 | São Manoel do Paraná | 0 | 0.77 | 0.71 | High and medium |
| 134 | Arapongas | 0 | 0.85 | 0.75 | High and medium |
| 135 | São Tomé | 0 | 0.88 | 0.71 | High and medium |
| 136 | Quinta do Sol | 2 | 0.99 | 0.68 | High and medium |
| 137 | Cornélio Procópio | 0 | 0.82 | 0.73 | High and medium |
| 138 | Novo Itacolomi | 0 | 0.88 | 0.69 | High and medium |
| 139 | Fênix | 1 | 0.95 | 0.74 | High and medium |
| 140 | Engenheiro Beltrão | 0 | 0.97 | 0.68 | High and medium |
| 141 | Quarto Centenário | 0 | 0.90 | 0.72 | High and medium |
| 142 | Cândido de Abreu | 9 | 0.83 | 0.57 | High and medium |
| 143 | Jussara | 0 | 0.98 | 0.71 | High and medium |
| 144 | Ivatuba | 0 | 1.00 | 0.66 | High and medium |
| 145 | Marumbi | 0 | 0.97 | 0.74 | High and medium |
| 146 | Londrina | 60 | 0.87 | 0.69 | High and medium |
| 147 | Mato Rico | 5 | 0.75 | 0.56 | High and medium |
| 148 | Cambé | 0 | 0.84 | 0.73 | High and medium |
| 149 | Nova América da Colina | 0 | 0.91 | 0.71 | High and medium |
| 150 | Floraí | 0 | 0.95 | 0.75 | High and medium |
| 151 | Curiúva | 1 | 0.90 | 0.48 | High and medium |
| 152 | Santo Antônio do Paraíso | 0 | 0.78 | 0.67 | High and medium |
| 153 | Borrazópolis | 2 | 0.79 | 0.73 | High and medium |
| 154 | Ubiratã | 10 | 0.86 | 0.75 | High and medium |
| 155 | Tamarana | 0 | 0.82 | 0.59 | High and medium |
| 156 | Rio Bom | 0 | 0.83 | 0.72 | High and medium |
| 157 | Tuneiras do Oeste | 0 | 0.84 | 0.74 | High and medium |
| 158 | Ipiranga | 0 | 0.80 | 0.55 | High and medium |
| 159 | Floresta | 0 | 1.00 | 0.68 | High and medium |
| 160 | Apucarana | 33 | 0.86 | 0.71 | High and medium |
| 161 | Godoy Moreira | 1 | 0.98 | 0.71 | High and medium |
| 162 | Santa Cecília do Pavão | 0 | 0.94 | 0.74 | High and medium |
| 163 | Figueira | 1 | 0.98 | 0.72 | High and medium |
| 164 | Pérola | 0 | 0.64 | 0.92 | Medium and high |
| 165 | Brasilândia do Sul | 0 | 0.62 | 0.80 | Medium and high |
| 166 | Assis Chateaubriand | 0 | 0.61 | 0.81 | Medium and high |
| 167 | Jardim Olinda | 2 | 0.64 | 0.88 | Medium and high |
| 168 | Formosa do Oeste | 11 | 0.74 | 0.82 | Medium and high |
| 169 | Tupãssi | 0 | 0.54 | 0.83 | Medium and high |
| 170 | Toledo | 0 | 0.50 | 0.76 | Medium and high |
| 171 | Francisco Alves | 2 | 0.65 | 0.86 | Medium and high |
| 172 | Cafelândia | 0 | 0.54 | 0.82 | Medium and high |
| 173 | Ribeirão Claro | 7 | 0.70 | 0.79 | Medium and high |
| 174 | Ibiporã | 0 | 0.74 | 0.76 | Medium and high |
| 175 | Amaporã | 1 | 0.74 | 0.83 | Medium and high |
| 176 | Leópolis | 0 | 0.69 | 0.80 | Medium and high |
| 177 | Guaporema | 0 | 0.70 | 0.87 | Medium and high |
| 178 | Paranapoema | 0 | 0.72 | 0.84 | Medium and high |
| 179 | Porto Rico | 61 | 0.70 | 0.83 | Medium and high |
| 180 | Corbélia | 1 | 0.48 | 0.76 | Medium and high |
| 181 | Maripá | 0 | 0.59 | 0.75 | Medium and high |
| 182 | Guaíra | 0 | 0.74 | 0.75 | Medium and high |
| 183 | São José da Boa Vista | 31 | 0.64 | 0.81 | Medium and high |
| 184 | Cafezal do Sul | 0 | 0.53 | 0.87 | Medium and high |
| 185 | São Jorge do Patrocínio | 3 | 0.71 | 0.83 | Medium and high |
| 186 | Santo Antônio do Caiuá | 1 | 0.75 | 0.85 | Medium and high |
| 187 | Primeiro de Maio | 0 | 0.55 | 0.82 | Medium and high |
| 188 | Xambrê | 0 | 0.68 | 0.91 | Medium and high |
| 189 | Uraí | 0 | 0.74 | 0.82 | Medium and high |
| 190 | Santana do Itararé | 115 | 0.59 | 0.87 | Medium and high |
| 191 | Iracema do Oeste | 0 | 0.72 | 0.83 | Medium and high |
| 192 | Nova Aurora | 72 | 0.72 | 0.79 | Medium and high |
| 193 | Alto Piquiri | 0 | 0.68 | 0.83 | Medium and high |
| 194 | Siqueira Campos | 65 | 0.68 | 0.85 | Medium and high |
| 195 | Perobal | 0 | 0.69 | 0.83 | Medium and high |
| 196 | Braganey | 0 | 0.47 | 0.77 | Medium and high |
| 197 | Carlópolis | 49 | 0.72 | 0.89 | Medium and high |
| 198 | Marilena | 24 | 0.75 | 0.86 | Medium and high |
| 199 | Sertaneja | 0 | 0.58 | 0.77 | Medium and high |
| 200 | Altônia | 3 | 0.74 | 0.86 | Medium and high |
| 201 | São Pedro do Paraná | 16 | 0.72 | 0.88 | Medium and high |
| 202 | Wenceslau Braz | 37 | 0.64 | 0.85 | Medium and high |
| 203 | Jesuítas | 6 | 0.70 | 0.81 | Medium and high |
| 204 | Salto do Itararé | 35 | 0.55 | 0.88 | Medium and high |
| 205 | Jataizinho | 0 | 0.73 | 0.77 | Medium and high |
| 206 | Itaguajé | 0 | 0.75 | 0.89 | Medium and high |
| 207 | Jacarezinho | 5 | 0.67 | 0.75 | Medium and high |
| 208 | Mirador | 0 | 0.72 | 0.84 | Medium and high |
| 209 | Santa Cruz de Monte Castelo | 31 | 0.74 | 0.80 | Medium and high |
| 210 | Sertanópolis | 0 | 0.69 | 0.76 | Medium and high |
| 211 | Iporã | 1 | 0.57 | 0.86 | Medium and high |
| 212 | Diamante do Norte | 87 | 0.70 | 0.82 | Medium and high |
| 213 | Marmeleiro | 0 | 0.01 | 0.76 | Low and high |
| 214 | Verê | 0 | 0.00 | 0.79 | Low and high |
| 215 | Flor da Serra do Sul | 0 | 0.01 | 0.80 | Low and high |
| 216 | Bom Sucesso do Sul | 0 | 0.02 | 0.78 | Low and high |
| 217 | Nova Prata do Iguaçu | 0 | 0.01 | 0.78 | Low and high |
| 218 | Sulina | 0 | 0.06 | 0.78 | Low and high |
| 219 | Dois Vizinhos | 0 | 0.00 | 0.77 | Low and high |
| 220 | Cruzeiro do Iguaçu | 0 | 0.00 | 0.80 | Low and high |
| 221 | Boa Esperança do Iguaçu | 0 | 0.00 | 0.78 | Low and high |
| 222 | Saudade do Iguaçu | 0 | 0.19 | 0.76 | Low and high |
| 223 | Barracão | 0 | 0.02 | 0.77 | Low and high |
| 224 | São João | 0 | 0.02 | 0.78 | Low and high |
| 225 | Itapejara D'Oeste | 0 | 0.01 | 0.81 | Low and high |
| 226 | Antônio Olinto | 0 | 0.25 | 0.45 | Medium and medium |
| 227 | Virmond | 2 | 0.48 | 0.59 | Medium and medium |
| 228 | Rio Branco do Sul | 0 | 0.36 | 0.37 | Medium and medium |
| 229 | Laranjeiras do Sul | 9 | 0.34 | 0.65 | Medium and medium |
| 230 | Marechal Cândido Rondon | 0 | 0.56 | 0.68 | Medium and medium |
| 231 | Alvorada do Sul | 0 | 0.62 | 0.73 | Medium and medium |
| 232 | Palmital | 0 | 0.73 | 0.71 | Medium and medium |
| 233 | Turvo | 0 | 0.42 | 0.32 | Medium and medium |
| 234 | Cerro Azul | 4 | 0.41 | 0.30 | Medium and medium |
| 235 | Diamante do Sul | 0 | 0.59 | 0.65 | Medium and medium |
| 236 | Faxinal | 2 | 0.72 | 0.67 | Medium and medium |
| 237 | Ramilândia | 0 | 0.30 | 0.65 | Medium and medium |
| 238 | Campo Largo | 0 | 0.33 | 0.35 | Medium and medium |
| 239 | Arapoti | 0 | 0.60 | 0.56 | Medium and medium |
| 240 | Porto Barreiro | 0 | 0.27 | 0.62 | Medium and medium |
| 241 | São Mateus do Sul | 0 | 0.27 | 0.54 | Medium and medium |
| 242 | Laranjal | 0 | 0.64 | 0.67 | Medium and medium |
| 243 | Ventania | 0 | 0.68 | 0.50 | Medium and medium |
| 244 | Vera Cruz do Oeste | 6 | 0.31 | 0.75 | Medium and medium |
| 245 | Pato Bragado | 0 | 0.43 | 0.69 | Medium and medium |
| 246 | Cambará | 0 | 0.51 | 0.75 | Medium and medium |
| 247 | Guaraniaçu | 0 | 0.39 | 0.58 | Medium and medium |
| 248 | Espigão Alto do Iguaçu | 0 | 0.31 | 0.54 | Medium and medium |
| 249 | Porto Amazonas | 0 | 0.25 | 0.64 | Medium and medium |
| 250 | Iguatu | 0 | 0.59 | 0.73 | Medium and medium |
| 251 | Missal | 4 | 0.31 | 0.71 | Medium and medium |
| 252 | Rancho Alegre | 0 | 0.67 | 0.75 | Medium and medium |
| 253 | Diamante D'Oeste | 0 | 0.33 | 0.65 | Medium and medium |
| 254 | Marquinho | 1 | 0.67 | 0.64 | Medium and medium |
| 255 | Candói | 2 | 0.42 | 0.6 | Medium and medium |
| 256 | Santa Maria do Oeste | 39 | 0.46 | 0.56 | Medium and medium |
| 257 | Santa Helena | 0 | 0.34 | 0.70 | Medium and medium |
| 258 | Palotina | 0 | 0.63 | 0.71 | Medium and medium |
| 259 | Campina do Simão | 0 | 0.30 | 0.45 | Medium and medium |
| 260 | Cantagalo | 0 | 0.54 | 0.58 | Medium and medium |
| 261 | Nova Santa Rosa | 0 | 0.63 | 0.67 | Medium and medium |
| 262 | Ortigueira | 20 | 0.66 | 0.58 | Medium and medium |
| 263 | Tibagi | 9 | 0.64 | 0.53 | Medium and medium |
| 264 | Goioxim | 3 | 0.52 | 0.51 | Medium and medium |
| 265 | Santa Mariana | 0 | 0.72 | 0.66 | Medium and medium |
| 266 | Palmeira | 0 | 0.31 | 0.66 | Medium and medium |
| 267 | Mercedes | 0 | 0.62 | 0.74 | Medium and medium |
| 268 | Ponta Grossa | 0 | 0.58 | 0.60 | Medium and medium |
| 269 | São Pedro do Iguaçu | 0 | 0.37 | 0.73 | Medium and medium |
| 270 | Teixeira Soares | 16 | 0.62 | 0.47 | Medium and medium |
| 271 | Foz do Jordão | 0 | 0.27 | 0.49 | Medium and medium |
| 272 | Rosário do Ivaí | 148 | 0.66 | 0.67 | Medium and medium |
| 273 | Fernandes Pinheiro | 0 | 0.36 | 0.57 | Medium and medium |
| 274 | Campo Magro | 0 | 0.26 | 0.41 | Medium and medium |
| 275 | Anahy | 0 | 0.62 | 0.74 | Medium and medium |
| 276 | Campo Bonito | 0 | 0.40 | 0.62 | Medium and medium |
| 277 | Campina da Lagoa | 28 | 0.62 | 0.71 | Medium and medium |
| 278 | Doutor Ulysses | 1 | 0.32 | 0.32 | Medium and medium |
| 279 | Itaipulândia | 0 | 0.29 | 0.65 | Medium and medium |
| 280 | Irati | 2 | 0.47 | 0.51 | Medium and medium |
| 281 | Itaperuçu | 0 | 0.40 | 0.25 | Medium and medium |
| 282 | São Jerônimo da Serra | 1 | 0.69 | 0.59 | Medium and medium |
| 283 | Porecatu | 0 | 0.67 | 0.75 | Medium and medium |
| 284 | Ouro Verde do Oeste | 0 | 0.44 | 0.71 | Medium and medium |
| 285 | São José das Palmeiras | 0 | 0.38 | 0.72 | Medium and medium |
| 286 | Pitanga | 0 | 0.72 | 0.62 | Medium and medium |
| 287 | Itambaracá | 0 | 0.72 | 0.72 | Medium and medium |
| 288 | Castro | 0 | 0.25 | 0.49 | Medium and medium |
| 289 | Reserva | 0 | 0.59 | 0.51 | Medium and medium |
| 290 | Andirá | 0 | 0.73 | 0.73 | Medium and medium |
| 291 | São João do Triunfo | 0 | 0.27 | 0.46 | Medium and medium |
| 292 | Imbaú | 0 | 0.46 | 0.42 | Medium and medium |
| 293 | Grandes Rios | 6 | 0.66 | 0.70 | Medium and medium |
| 294 | Querência do Norte | 74 | 0.66 | 0.74 | Medium and medium |
| 295 | Imbituva | 44 | 0.73 | 0.54 | Medium and medium |
| 296 | Carambeí | 0 | 0.38 | 0.73 | Medium and medium |
| 297 | Sengés | 0 | 0.30 | 0.47 | Medium and medium |
| 298 | Nova Laranjeiras | 2 | 0.43 | 0.52 | Medium and medium |
| 299 | Altamira do Paraná | 1 | 0.53 | 0.63 | Medium and medium |
| 300 | Mauá da Serra | 0 | 0.45 | 0.60 | Medium and medium |
| 301 | Quatro Pontes | 0 | 0.59 | 0.74 | Medium and medium |
| 302 | Boa Ventura de São Roque | 1 | 0.62 | 0.63 | Medium and medium |
| 303 | Roncador | 6 | 0.75 | 0.62 | Medium and medium |
| 304 | Nova Cantu | 0 | 0.47 | 0.67 | Medium and medium |
| 305 | Entre Rios do Oeste | 0 | 0.40 | 0.65 | Medium and medium |
| 306 | Rebouças | 15 | 0.29 | 0.53 | Medium and medium |
| 307 | Telêmaco Borba | 1 | 0.65 | 0.12 | Medium and low |
| 308 | Piên | 0 | 0.09 | 0.42 | Low and medium |
| 309 | Araucária | 1 | 0.23 | 0.72 | Low and medium |
| 310 | Bela Vista da Caroba | 0 | 0.03 | 0.75 | Low and medium |
| 311 | Rio Bonito do Iguaçu | 5 | 0.25 | 0.51 | Low and medium |
| 312 | Jaguariaíva | 7 | 0.13 | 0.48 | Low and medium |
| 313 | Mangueirinha | 0 | 0.04 | 0.61 | Low and medium |
| 314 | Curitiba | 16 | 0.20 | 0.57 | Low and medium |
| 315 | Rio Negro | 0 | 0.21 | 0.38 | Low and medium |
| 316 | Capanema | 0 | 0.02 | 0.66 | Low and medium |
| 317 | Quitandinha | 0 | 0.15 | 0.57 | Low and medium |
| 318 | Cascavel | 7 | 0.22 | 0.65 | Low and medium |
| 319 | Três Barras do Paraná | 0 | 0.09 | 0.65 | Low and medium |
| 320 | União da Vitória | 0 | 0.17 | 0.29 | Low and medium |
| 321 | Pranchita | 0 | 0.04 | 0.62 | Low and medium |
| 322 | Medianeira | 0 | 0.22 | 0.75 | Low and medium |
| 323 | Pinhão | 0 | 0.14 | 0.36 | Low and medium |
| 324 | Santa Tereza do Oeste | 0 | 0.21 | 0.67 | Low and medium |
| 325 | Cruz Machado | 0 | 0.11 | 0.28 | Low and medium |
| 326 | Coronel Domingos Soares | 0 | 0.04 | 0.29 | Low and medium |
| 327 | Francisco Beltrão | 0 | 0.01 | 0.66 | Low and medium |
| 328 | Enéas Marques | 0 | 0.00 | 0.68 | Low and medium |
| 329 | Capitão Leônidas Marques | 0 | 0.07 | 0.70 | Low and medium |
| 330 | Salgado Filho | 0 | 0.04 | 0.66 | Low and medium |
| 331 | Coronel Vivida | 0 | 0.01 | 0.71 | Low and medium |
| 332 | Rio Azul | 13 | 0.23 | 0.44 | Low and medium |
| 333 | Porto Vitória | 0 | 0.20 | 0.32 | Low and medium |
| 334 | Guarapuava | 0 | 0.24 | 0.44 | Low and medium |
| 335 | Ampére | 0 | 0.04 | 0.69 | Low and medium |
| 336 | Realeza | 0 | 0.04 | 0.73 | Low and medium |
| 337 | Piraquara | 0 | 0.11 | 0.47 | Low and medium |
| 338 | Tijucas do Sul | 0 | 0.03 | 0.29 | Low and medium |
| 339 | São Jorge D'Oeste | 0 | 0.00 | 0.70 | Low and medium |
| 340 | Campo do Tenente | 0 | 0.21 | 0.44 | Low and medium |
| 341 | Balsa Nova | 0 | 0.14 | 0.67 | Low and medium |
| 342 | Piraí do Sul | 0 | 0.17 | 0.59 | Low and medium |
| 343 | Clevelândia | 0 | 0.00 | 0.56 | Low and medium |
| 344 | Matelândia | 0 | 0.11 | 0.38 | Low and medium |
| 345 | Chopinzinho | 0 | 0.15 | 0.67 | Low and medium |
| 346 | Foz do Iguaçu | 0 | 0.13 | 0.50 | Low and medium |
| 347 | Boa Vista da Aparecida | 2 | 0.08 | 0.70 | Low and medium |
| 348 | Santa Izabel do Oeste | 0 | 0.01 | 0.75 | Low and medium |
| 349 | Salto do Lontra | 0 | 0.00 | 0.75 | Low and medium |
| 350 | Fazenda Rio Grande | 0 | 0.18 | 0.63 | Low and medium |
| 351 | Vitorino | 0 | 0.01 | 0.72 | Low and medium |
| 352 | Nova Esperança do Sudoeste | 0 | 0.00 | 0.69 | Low and medium |
| 353 | Lapa | 0 | 0.23 | 0.55 | Low and medium |
| 354 | São Miguel do Iguaçu | 0 | 0.12 | 0.62 | Low and medium |
| 355 | Ibema | 0 | 0.17 | 0.62 | Low and medium |
| 356 | Pinhal do São Bento | 0 | 0.04 | 0.65 | Low and medium |
| 357 | Pérola D'Oeste | 0 | 0.01 | 0.66 | Low and medium |
| 358 | Pinhais | 0 | 0.21 | 0.55 | Low and medium |
| 359 | Santo Antônio do Sudoeste | 0 | 0.04 | 0.72 | Low and medium |
| 360 | Palmas | 0 | 0.00 | 0.45 | Low and medium |
| 361 | Mariópolis | 0 | 0.00 | 0.75 | Low and medium |
| 362 | Paula Freitas | 0 | 0.22 | 0.50 | Low and medium |
| 363 | Mallet | 0 | 0.17 | 0.35 | Low and medium |
| 364 | Agudos do Sul | 0 | 0.02 | 0.44 | Low and medium |
| 365 | Quedas do Iguaçu | 0 | 0.16 | 0.52 | Low and medium |
| 366 | Reserva do Iguaçu | 0 | 0.11 | 0.32 | Low and medium |
| 367 | Honório Serpa | 0 | 0.00 | 0.61 | Low and medium |
| 368 | São José dos Pinhais | 0 | 0.09 | 0.37 | Low and medium |
| 369 | Mandirituba | 0 | 0.07 | 0.46 | Low and medium |
| 370 | Santa Lúcia | 4 | 0.11 | 0.74 | Low and medium |
| 371 | Pato Branco | 0 | 0.02 | 0.74 | Low and medium |
| 372 | Bom Jesus do Sul | 0 | 0.04 | 0.73 | Low and medium |
| 373 | Renascença | 0 | 0.01 | 0.71 | Low and medium |
| 374 | Manfrinópolis | 0 | 0.02 | 0.60 | Low and medium |
| 375 | Contenda | 0 | 0.19 | 0.70 | Low and medium |
| 376 | Santa Terezinha de Itaipu | 0 | 0.13 | 0.72 | Low and medium |
| 377 | Almirante Tamandaré | 3 | 0.05 | 0.50 | Low and medium |
| 378 | Planalto | 0 | 0.02 | 0.73 | Low and medium |
| 379 | Catanduvas | 0 | 0.17 | 0.63 | Low and medium |
| 380 | Colombo | 0 | 0.12 | 0.59 | Low and medium |
| 381 | Lindoeste | 0 | 0.16 | 0.60 | Low and medium |
| 382 | Paulo Frontin | 0 | 0.24 | 0.53 | Low and medium |
| 383 | Guaraqueçaba | 0 | 0.00 | 0.03 | Low and low |
| 384 | General Carneiro | 0 | 0.02 | 0.21 | Low and low |
| 385 | Morretes | 0 | 0.01 | 0.05 | Low and low |
| 386 | Antonina | 0 | 0.01 | 0.04 | Low and low |
| 387 | Campina Grande do Sul | 0 | 0.08 | 0.20 | Low and low |
| 388 | Bituruna | 0 | 0.11 | 0.21 | Low and low |
| 389 | Pontal do Paraná | 0 | 0.02 | 0.06 | Low and low |
| 390 | Bocaiúva do Sul | 1 | 0.10 | 0.21 | Low and low |
| 391 | Inácio Martins | 0 | 0.04 | 0.14 | Low and low |
| 392 | Tunas do Paraná | 0 | 0.12 | 0.05 | Low and low |
| 393 | Paranaguá | 0 | 0.01 | 0.08 | Low and low |
| 394 | Matinhos | 0 | 0.01 | 0.05 | Low and low |
| 395 | Quatro Barras | 1 | 0.09 | 0.20 | Low and low |
| 396 | Adrianópolis | 0 | 0.09 | 0.11 | Low and low |
| 397 | Serranópolis do Iguaçu | 0 | 0.00 | 0.23 | Low and low |
| 398 | Guaratuba | 0 | 0.02 | 0.03 | Low and low |
| 399 | Céu Azul | 12 | 0.19 | 0.22 | Low and low |
