# Supplementary material for: Spatial prediction of risk areas for vector transmission of Trypanosoma cruzi in the State of Paraná, southern Brazil
Source: PLoS Negl Trop Dis. 2018 Oct 26;12(10):e0006907. doi: 10.1371/journal.pntd.0006907 (PMC6221357; doi:10.1371/journal.pntd.0006907)
Supplement: S4 Table — We used the following algorithms: Bioclim, Gower, Maxent, and numbers from 1 to 10 are the model replicates. SD—Standard deviation. (DOCX) [file pntd.0006907.s004.docx]

**S4 Table. The True Skill Statistic (TSS) values of climate-based models and landscape-based models.** Bioclim, Gower, Maxent, SVM used algorithms. Numbers 1 to 10 are the replications. SD - Standard deviation.

|  | **Bioclim** | **Gower** | **Maxent** | **SVM** | **Bioclim** | **Gower** | **Maxent** | **SVM** |
| --- | --- | --- | --- | --- | --- | --- | --- | --- |
| 1 | 0.565 | 0.556 | 0.563 | 0.546 | 0.484 | 0.499 | 0.614 | 0.624 |
| 2 | 0.592 | 0.607 | 0.646 | 0.617 | 0.430 | 0.429 | 0.559 | 0.561 |
| 3 | 0.653 | 0.674 | 0.717 | 0.716 | 0.570 | 0.584 | 0.635 | 0.599 |
| 4 | 0.629 | 0.638 | 0.679 | 0.646 | 0.526 | 0.541 | 0.664 | 0.622 |
| 5 | 0.637 | 0.718 | 0.718 | 0.655 | 0.588 | 0.591 | 0.675 | 0.643 |
| 6 | 0.665 | 0.627 | 0.670 | 0.640 | 0.559 | 0.574 | 0.633 | 0.686 |
| 7 | 0.624 | 0.658 | 0.643 | 0.587 | 0.566 | 0.603 | 0.673 | 0.639 |
| 8 | 0.580 | 0.589 | 0.656 | 0.684 | 0.521 | 0.531 | 0.589 | 0.669 |
| 9 | 0.586 | 0.566 | 0.669 | 0.655 | 0.481 | 0.507 | 0.615 | 0.576 |
| 10 | 0.601 | 0.593 | 0.623 | 0.626 | 0.613 | 0.610 | 0.585 | 0.580 |
| Mean | 0.613 | 0.623 | 0.658 | 0.637 | 0.534 | 0.547 | 0.624 | 0.620 |
| SD | 0.033 | 0.051 | 0.045 | 0.048 | 0.056 | 0.057 | 0.039 | 0.041 |
